# Peroxiredoxin 1 is a tumor-associated antigen in esophageal squamous cell carcinoma

**DOI:** 10.3892/or.2013.2714

**Published:** 2013-09-04

**Authors:** PENGFEI REN, HUA YE, LIPING DAI, MEI LIU, XINXIN LIU, YURONG CHAI, QING SHAO, YANG LI, NINGJING LEI, BO PENG, WU YAO, JIANYING ZHANG

**Affiliations:** 1 Henan Key Laboratory of Tumor Epidemiology and College of Public Health, Zhengzhou University, Zhengzhou, Henan 450052, P.R. China; 2 Department of Biological Sciences, The University of Texas at El Paso, El Paso, TX 79968, USA

**Keywords:** autoantibody, peroxiredoxin 1, esophageal squamous cell carcinoma, biomarker, immunodiagnosis

## Abstract

Peroxiredoxin 1 (Prdx1) is an antioxidant and plays an important role in H_2_O_2_-mediated cell signaling. We previously found that the expression level of Prdx1 was elevated in esophagus squamous cell carcinoma (ESCC) tissue using a proteomics approach. Since overexpressed protein can induce an autoimmune response, to further examine whether serum from ESCC patients exhibits immunoreactivity against Prdx1, autoantibody responses to Prdx1 were evaluated by ELISA, western blotting and indirect immunofluorescence assay in sera from patients with ESCC and normal individuals. Immunohistochemical study with tissue array slides and western blot analysis with cancer cell lines were also performed to analyze the protein expression profiles of Prdx1 in ESCC tissues and cancer cell lines. The results demonstrated that the positive rate of autoantibody against Prdx1 in ESCC sera was 13.2% (9/68), whereas this rate was 0% (0/89) in normal individuals. Data also showed that expression of Prdx1 was significantly increased in ESCC tissues when compared to expression in paired adjacent normal tissues (P<0.05). The data indicate that Prdx1 may contribute to malignant transformation of the esophagus, and may be used as a biomarker in the immunodiagnosis of ESCC.

## Introduction

Tumor-associated antigens (TAAs) are a category of proteins relevant to the occurrence, transformation and progression of cancer, which evoke an immune response and elicit autoantibodies to these antigens. TAAs and anti-TAA autoantibodies can be used as biomarkers for diagnosing cancer or predicting the prognosis of disease. Although there has been a rapid increase in the number of TAAs identified using proteomics approach or serological analysis of recombinant cDNA expression libraries (SEREX), a great percentage of these identified TAAs, however, were found to have no direct relevance to cancer. Thus, it is necessary to further evaluate and validate these candidate TAAs as diagnostic markers or therapeutic targets of immunotherapy (1). The humoral immune response of candidate TAAs can be detected by using immunoassays such as ELISA or western blotting in large scale sera from cancer patients and controls under different clinical conditions when using recombinant proteins as target antigens (2).

Peroxiredoxins (Prdxs) are a family of 22–27 kDa non-selenium-dependent glutathione peroxidases which destroy peroxides, organic hydroperoxides and peroxynitrite (3,4). Six isoforms of Prdxs have been identified in mammals, and are divided into 3 subclasses: typical 2-cysteine Prdxs (Prdx1-4), atypical 2-cysteine (Prdx5) and 1-cysterine Prdx (Prdx6) (5). They localize in different locations of the cell; Prdx1, Prdx2 and Prdx6 are localized to the cytoplasm, Prdx3 in the mitochondria, Prdx4 in the extracellular space, and Prdx5 in the mitochondria and peroxisomes (4). Prdx1 has been viewed as a tumor-suppressor as Prdx1-knockout mice exhibit a shortened life span due to the development of hemolytic anemia and cancer. One study demonstrated that Prdx1 inhibits the activation of oncogenes such as c-Abl and c-myc, and it can also be considered as a safeguard for the lipid phosphatase activity of PTEN, which is essential for its tumor-suppressive function (6).

Prdx1 was recently identified as a candidate esophageal squamous cell carcinoma (ESCC)-related TAA in a previous study using a proteomics approach. It was found that Prdx1 was overexpressed in ESCC tissues when compared to adjacent normal tissues (7,8), and the expression level of this protein was also elevated in other types of tumor tissues (9–16). In addition, it was found that Prdx1 induces the production of an autoantibody against this protein in the sera of patients with non-small cell lung cancer (NSCLC), but to date there is no report available regarding whether this protein induces an autoimmune response in ESCC. In order to further characterize and validate the identified tumor-associated protein Prdx1, recombinant Prdx1 protein was subsequently used as a target antigen to screen the anti-Prdx1 autoantibody in sera from patients with ESCC and normal individuals by ELISA and western blotting. Indirect immunofluorescence assay with cancer cell lines and immunohistochemistry with cancer tissue array slides were also performed to analyze the protein expression profiles of Prdx1 in cancer cells and tissues.

## Materials and methods

### Sera and general information

Sera from 68 patients with ESCC and 89 normal human sera (NHS) were obtained from the serum bank of the Cancer Autoimmunity and Epidemiology Research Laboratory at the University of Texas, El Paso (UTEP), which were originally provided by Dr X.-X. Peng of Sun Yat-sen University, Guangzhou, China. All ESCC cases were confirmed by histopathological diagnosis. All ESCC sera were collected at the time of initial cancer diagnosis, when the patients had not yet received any chemotherapy or radiation therapy. Normal human sera were collected during annual health examinations from individuals who had no obvious evidence of malignancy.

### Cell lines and cell extracts

Nine different tumor cell lines [human epidermoid carcinoma (Hep2), human hepatocellular carcinoma (HepG2), human hepatocellular carcinoma (SUN449), human breast cancer (SKBR3), human ovarian carcinoma (SKOV3), human lung epithelial adenocarcinoma (A549), human urinary bladder carcinoma (T24), human acute lymphoblastic leukemia (MOLT-4) and leukemia (KOPN63)] were obtained from the tumor cell bank of our laboratory and cultured following the specific protocol for each cell line. Cells grown in monolayers were solubilized in Laemmli’s sample buffer containing protease inhibitors after sonication. Solubilized lysates were briefly denatured before electrophoresis on SDS-polyacrylamide gels.

### Enzyme-linked immunosorbent assay (ELISA)

Standard protocol for ELISA was used as described in our previous study (12). In brief, a 96-well microtiter plate (Thermo Scientific, Waltham, MA, USA) was coated overnight at 4°C with recombinant Prdx1 protein (Abcam, Cambridge, MA, USA) at a final concentration of 0.5 μg/ml in phosphate-buffered saline (PBS). The antigen-coated wells were blocked with gelatin post-coating solution at room temperature for 2 h. Human sera diluted at 1:100 with serum diluent were incubated for 2 h at room temperature in the antigen-coated wells, followed by HRP-conjugated goat anti-human IgG (Santa Cruz Biotechnology, Inc., Santa Cruz, CA, USA). The substrate 2,2′-azino-bis-3-ethylbenzo-thiazoline-6-sulfonic acid (ABTS; Sigma-Aldrich, St. Louis, MO, USA) was used as the detecting reagent. The average optical density (OD) value at a wavelength of 405 nm was applied as data analysis. The cutoff value designating a positive reaction was the mean OD of 89 NHS + 3SD.

### Western blotting

Denatured recombinant Prdx1 protein and tumor cell lysates were electrophoresed on 12% SDS-PAGE and transferred to nitrocellulose membranes, respectively. After blocking in PBS with 5% non-fat milk and 0.05% Tween-20 for 1 h at room temperature, the nitrocellulose membranes were incubated overnight at 4°C with a 1:200 dilution of human sera, a 1:1,000 dilution of polyclonal anti-Prdx1 antibody (GeneTex Inc., Irvine, CA, USA) and a 1:500 dilution of monoclonal anti-β-actin antibody (Cell Signaling Technology, Inc., Danvers, MA, USA), separately. HRP-conjugated goat anti-human IgG, HRP-conjugated goat anti-rabbit IgG and HRP-conjugated goat anti-mouse IgG (Santa Cruz Biotechnology, Inc.) were applied as secondary antibodies at a 1:3,000 dilution. The ECL kit was used to detect immunoreactive bands according to the manufacturer’s instructions (Thermo Scientific).

### Absorption of antibodies with recombinant protein

The diluted human sera (1:80) were incubated with recombinant protein (final concentration of recombinant protein in the diluted human sera was 0.01 μg/μl) overnight at 4°C, and then centrifuged at 10,000 × g for 15 min. The supernatant was used for immunofluorescence assay.

### Indirect immunofluorescence assay (IIFA)

Hep-2 antigen substrate for the IIFA test system was incubated with a dilution of sera (1:80) and preabsorbed sera at 4°C overnight. FITC-conjugated goat anti-human IgG (Santa Cruz Biotechnology, Inc.) was used as the secondary antibody at a 1:100 dilution. A fluorescence microscope (Leica DM1000, Germany) was used for examination.

### Immunohistochemical (IHC) analysis of the tissue assay slides

ESCC tissue array slides with adjacent normal tissue controls (16 ESCC tissue specimens, 14 adjacent tissue specimens and 12 normal esophagus specimens) with information regarding clinical stages and pathological grades) were commercially purchased (US Biomax, Inc., Rockville, MD, USA), and used to detect the expression of Prdx1 protein. There were 7 cases containing completely self-paired ESCC, adjacent and normal specimens. Tissue array slides were deparaffinized with xylene and dehydrated with ethanol. Antigen retrieval was performed by microwave-heating methods in Trilogy™ pretreatment solution for 20 min. Avidin/biotin blocking solution was used to prevent nonspecific binding of the antibodies. The sections were incubated with polyclonal anti-Prdx1 antibody (1:50 dilution) for 1 h at room temperature. HRP detection system (HRP streptavidin labeled and polyvalent biotinylated linked) and DAB substrate kit were used as detecting reagents. After counterstaining with hematoxylin, the sections were dehydrated and mounted. The slides were observed using a microscope (Leica DM1000).

### Statistical analysis

The mean OD value of each group of patient sera was compared using the Mann-Whitney U test. The frequencies of antibodies to Prdx1 in each group of patient sera were compared using the χ^2^ test with Yate’s correction. Sensitivity and specificity were calculated as previously described (17). The expression profile of Prdx1 in the ESCC, adjacent and normal tissue groups was compared using χ^2^ test and Fisher’s exact test, whereas self-paired specimens of the different groups were compared using Cochran Q test, and 2 levels of significance (0.01 and 0.05) were used.

## Results

### Frequency and the titer of the autoantibody against Prdx1 in ESCC

**Figure 1:**
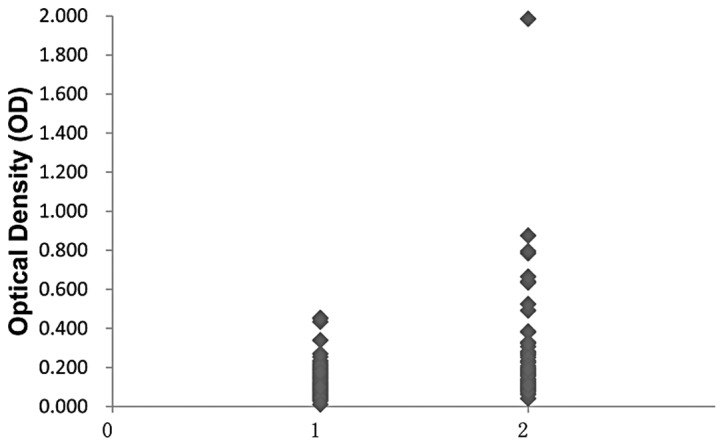
Titer of the autoantibody to Prdx1 in ESCC and normal human sera (NHS). The range of antibody titers to Prdx1 is expressed as the optical density (OD) obtained from ELISA. The y-axis represents the OD values; the x-axis represents serum samples: 1, NHS; 2, ESCC sera. The titer of anti-Prdx1 in ESCC was much higher than that in the NHS (P<0.01). Prdx1, peroxiredoxin 1; ESCC, esophageal squamous cell carcinoma.

**Figure 2:**
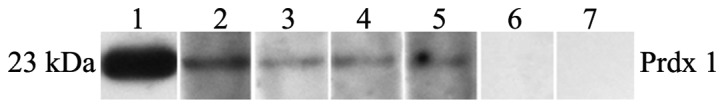
Western blot analysis of representative sera in ELISA. Lane 1, polyclonal anti-Prdx1 antibody used as the positive control; lanes 2–5, four representative ESCC sera which were positive in ELISA and also had strong reactivity with Prdx1 recombinant protein in the western blot analysis; lanes 6 and 7, two random normal human sera with negative reactivity to Prdx1 recombinant protein. Prdx1, peroxiredoxin 1; ESCC, esophageal squamous cell carcinoma.

The full length recombinant Prdx1 protein was used as the coating antigen in ELISA to detect the autoantibody against Prdx1 in sera from 68 patients with ESCC and 89 normal individuals. As demonstrated in Table I, the prevalence of an autoantibody against Prdx1 was 13.2% (9/68) in ESCC, which was significantly higher than that in the NHS (P<0.01). The titer of the autoantibody against Prdx1 is shown in Fig. 1. The average titer of the autoantibody against Prdx1 in ESCC was significantly higher than that in NHS (P<0.01). The ELISA result was also confirmed by western blot analysis. Fig. 2 shows four representative ESCC sera which were positive in ELISA, and also had strong reactivity in the western blot analysis.

### Perinuclear intense staining pattern in Hep-2 cells by indirect immunofluorescence assay with representative positive ESCC sera

**Figure 3:**
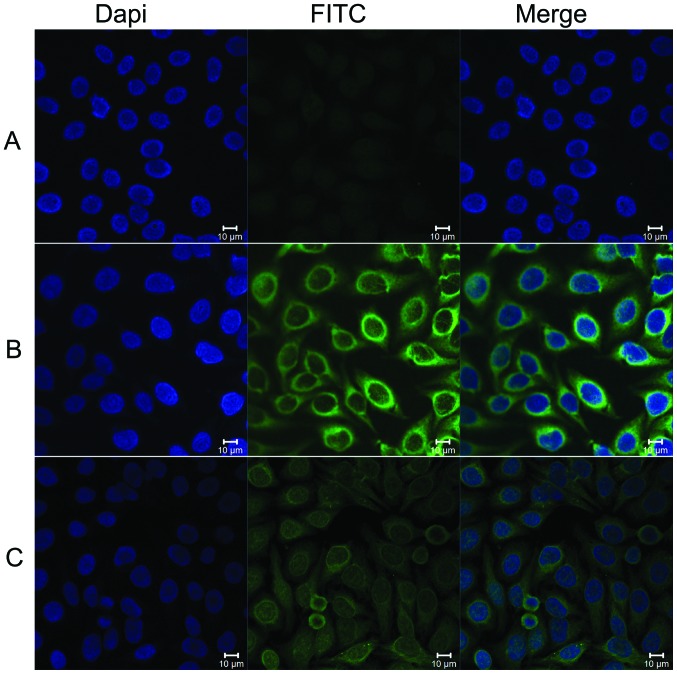
Representative immunofluorescence staining pattern of anti-Prdx1 antibody-positive ESCC serum. (A) A normal human serum sample used as a negative control. (B) A representative anti-Prdx1 antibody-positive ESCC serum sample demonstrating a perinuclear immunofluorescence staining pattern.(C) The same ESCC serum sample in B was pre-absorbed with recombinant Prdx1 protein, and subsequently utilized for immunofluorescence assay. The fluorescent signal was intensely reduced. Prdx1, peroxiredoxin 1; ESCC, esophageal squamous cell carcinoma.

To further confirm the specificity of an autoantibody response to Prdx1 in ESCC sera, ESCC sera with anti-Prdx1 positivity in ELISA were also examined by indirect immunofluoresence assay with commercially purchased Hep-2 cell slides. As shown in Fig. 3, a representative ESCC serum sample with anti-Prdx1 antibody positivity in ELISA had a cytoplasmic staining pattern with more intense staining in the perinuclear regions. The fluorescent signal was significantly reduced after being preabsorbed with the anti-Prdx1 antibobies with recombinant Prdx1 protein in the same serum.

### Expression of Prdx1 in ESCC tissues by immunohistochemistry with tissue array

**Figure 4:**
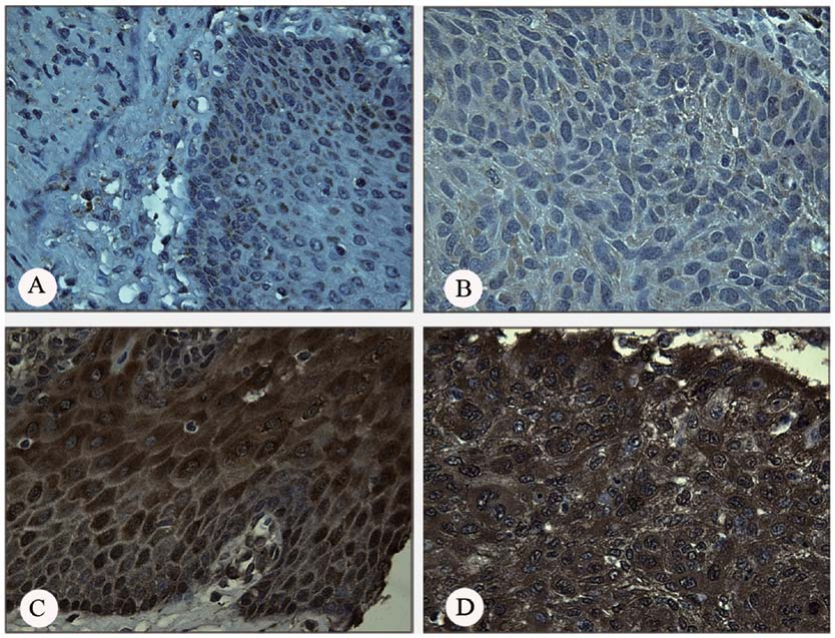
Expression of Prdx1 in ESCC, adjacent ESCC and normal tissues by immunohistochemistry. The polyclonal anti-Prdx1 antibody was used as the primary antibody to detect the expression of Prdx1 in ESCC, adjacent ESCC and normal esophagus tissues. (A) A representative normal esophageal tissue with anti-Prdx-negative staining (magnification, ×40). (B) A representative adjacent ESCC tissue with anti-Prdx1-negative staining (magnification, ×40). (C) A representative adjacent ESCC tissue with strong positive staining (magnification, ×40). (D) A representative ESCC tissue with strong positive staining (magnification, ×40). Prdx1, peroxiredoxin 1; ESCC, esophageal squamous cell carcinoma.

The expression profile of Prdx1 in ESCC, adjacent and normal esophagus tissues was examined by immunohistochemistry with tissue array slides. Tissue array slides containing 16 ESCC tissue specimens, 14 adjacent tissue specimens and 12 normal esophagus specimens, were commercially available for this study. The polyclonal anti-Prdx1 antibody was used as the primary antibody to detect the expression of Prdx1 in these tissues. As shown in Table II, the result indicated that there was an increased frequency of Prdx1 overexpression in ESCC tissues (100%, 16/16) compared to the adjacent carcinoma tissues (64.3%, 9/14) or normal tissues (50%, 6/12). The frequency of Prdx1 expression in ESCC tissues was significantly higher than that in the adjacent tissues (P<0.01) and normal tissues (P<0.05). Among the 7 self-paired cases (Table III), the frequency of Prdx1 was significantly higher in the ESCC tissues (7/7) than that in the adjacent carcinoma tissues (3/7) and in the normal tissues (3/7) (P<0.05). Fig. 4 shows a representative positive and negative immunostaining pattern of Prdx1 in ESCC and normal tissue. Due to the small sample size of the tissues examined, it was not possible to establish a statistical correlation between Prdx1 expression and clinical stage in the present study.

### Overexpression of Prdx1 in different cancer cell lines

**Figure 5:**
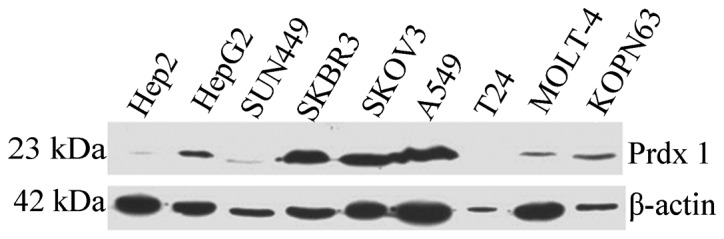
Nine types of tumor cell lines were analyzed by western blotting. The polyclonal anti-Prdx1 antibody was used as a probe. HepG2, SKBR3, SKOV3 and A549 cell lines showed strong reactivity to anti-Prdx1. The SUN449 cell line showed a moderate reactivity, while Hep2, MOLT-4 and KOPN63 cell lines showed weak reactivity. The T24 cell line was completely negative to the anti-Prdx1 antibody. Prdx1, peroxiredoxin 1.

To explore the expression level of Prdx1 in different types of cancers, 9 cancer cell lines (Hep2, HepG2, SUN449, SKBR3, SKOV3, A549, T24, MOLT-4 and KOPN63) were analyzed by western blotting. The polyclonal anti-Prdx1 antibody was used as a probe for this study. As shown in Fig. 5, based on the expression level of internal control β-actin, several human cancer cell lines such as HepG2 (hepatocellular carcinoma), SKBR3 (breast cancer), SKOV3 (ovarian carcinoma) and A549 (lung epithelial adenocarcinoma) exhibited a strong reactivity to anti-Prdx1. The SUN449 (hepatocellular carcinoma) cell line showed moderate reactivity, while Hep2 (epidermoid carcinoma), MOLT-4 (acute lymphoblastic leukemia) and KOPN63 (leukemia) cell lines exhibited weak reactivity. The T-24 (urinary bladder carcinoma) cell line was completely negative to the anti-Prdx1 antibody.

## Discussion

The family of Prdxs is one of the 3 major peroxidase consisting of catalase, Prdx and glutathione peroxidase, which function as scavengers of H_2_O_2_(5). Under small amounts of cellular H_2_O_2_, Prdx1 decomposes peroxides more efficiently than catalases due to its high affinity and wide cellular distribution, but it becomes over-oxidized under condition of high levels of H_2_O_2_, while catalases scavenge H_2_O_2_ rapidly and efficiently in this case (5). It has been shown that Prdxs and catalases function as peroxidases sequentially but not synergistically. Prdx1 modulates cell signaling pathways not only by influencing the intracellular levels of reactive oxygen species (ROS) and deactivating MAPK phosphatases indirectly induced by ROS, which results in c-Jun N-terminal kinase (JNK) activation, but also by directly interacting and inhibiting stress protein kinases such as c-Abl and JNK (18–20). Prdx1 was originally identified as a tumor-suppressor (6,20,21) based on the observation that the formation of sarcomas and blood malignancies was increased in Prdx1 gene knockout mice (21). Although the function of Prdx1 in the process of carcinogenesis is still not clear, various mechanisms have been recently proposed and verified in certain types of cancer. In cancer cells, the cellular level of H_2_O_2_ is abnormally high (22). Under a high level of H_2_O_2_, Prdx1 is over-oxidized and shifts the function from peroxidase to a chaperone (23). Over-oxidized Prdx1 can result in oligomerization and in losing its peroxidase activity, whereas it functions as a chaperone and regulates cell signaling via interacting with signaling proteins. Then it may lose the ability to form a complex with signaling partners such as the kinases c-Ab1 (20), JNK (9), the oncoprotein c-myc (24) and the phosphatase PTEN (6), resulting in activation of c-Ab1, JNK, c-myc and inactivation of PTEN’s phosphatase activity. Under normal conditions, Prdx1 interacts with the SH3 and kinase domains of c-Abl tyrosine kinase, thereby inhibiting the activity of c-Abl kinase (20). Under increased H_2_O_2_ stress, over-oxidized Prdx1 loses the ability to interact with these domains, resulting in the activation of c-Abl kinase (20). Prdx1 regulates c-myc signaling by binding to the myc box II, which is a highly conserved region of c-myc, inducing inhibition of c-myc which then causes a broad but selective loss of c-myc target gene regulation (24,25). Prdx1 regulates the tumor-suppressive function of PTEN by forming a complex with it, and inhibits inactivation of the lipid phosphatase of PTEN induced by H_2_O_2_. As two of its cysteine domains form a disulfide bond after oxidation, over-oxidized Prdx1 can lose the ability to combine with PTEN, resulting in hyperactive Akt signaling and oncogenesis (6). In addition, in prostate cancer, Prdx1 was found to be secreted into the extracellular location and to interact with Toll-like receptor 4 (TLR4), subsequently promoting angiogenesis and VEGF production, which eventually stimulated TLR4 and VEGF-dependent endothelial cell proliferation, migration and differentiation (15).

The present study revealed that there was a high level of expression of Prdx1 in the liver cancer cell line HepG2, breast, ovarian and lung cancer cell lines (SKBR3, SKOV3 and A549), and relatively weak expression in another type of liver cancer cell line (SUN449) and in laryngeal cancer (Hep2), acute lymphoblastic leukemia (MOLT-4) and lymphoma leukemia (KOPN63) cell lines. Our results also indicate that Prdx1 was not only overexpressed in certain solid tumors, but also in leukemia, suggesting the high relevance of Prdx1 with malignancy. The elevated expression of Prdx1 was also reported in numerous types of cancers by other groups (7,10,14,16). In the present study, the expression profile of Prdx1 in ESCC, adjacent and normal tissues was examined and evaluated by immunohistochemistry with tissue array slides. The expression level of Prdx1 was highly elevated in ESCC tissues when compared to adjacent and normal tissues. The data of paired ESCC with adjacent and normal tissues provided a similar result confirming that Prdx1 was overexpressed in cancer tissues while the paired samples were at a lower level. Hoshino *et al*(7) found that Prdx1 was overexpressed in 90% of the examined 114 ESCC samples. Due to the small sample size in the present study, it could not be determined whether the expression level was correlated with the pathological classification. However, Hoshino *et al*(7) showed that the expression level of Prdx1 was inversely correlated with depth of invasion and stage, and reduced expression predicted shorter overall survival. While comparing the expression profile of Prdx1 in ESCC tissues with other tumor types, there may be various significant differences which are notable. Prdx1 expression was increased in lung (10,11), liver (12), gallbladder (13), bladder (14), prostate (15) and ovarian cancer (16,26), and a high level of Prdx1 expression was significantly correlated with tumor grade and clinical stage in some types of tumors such as non-small cell lung cancer (NSCLC), gallbladder cancer and cholangiocarcinoma (10,12,13), but there was no correlation noted in ovarian carcinoma (16). Overexpression of Prdx1 was found to be correlated with overall survival and prognosis. A high level of Prdx1 expression was significantly correlated with poor overall survival in most reported cancers, but inversely reduced Prdx1 expression was correlated with reduced overall survival and poor clinical outcome in cholangiocarcinoma, suggesting that Prdx1 is a valuable prognostic marker in predicting the outcome, recurrence and overall survival in patients with certain types of tumors.

An anticancer agent, FK228, inhibits the growth of esophageal squamous cell cancer and induces apoptosis in part through Prdx1 activation. The sensitization of ESCC cells to FK228 was downregulated after silencing of Prdx1 gene expression (27). In prostate cancer, reduction in Prdx1 expression was found to lead to reduced tumor vasculature formation, and further inhibition of tumor growth (15). Therefore, the tumor growth and augmentation of radio-sensitivity by decreasing Prdx1 expression in lung cancer cell lines was inhibited (28). Taken together, these results also indicate that Prdx1 may be a potential therapeutic target for ESCC and other cancers.

To date, an autoimmune response to Prdx1 in ESCC has not been reported. A study from Korea showed that the positive rate of autoantibody against Prdx1 was 47.0% in 53 sera from NSCLC patients by western blot analysis, whereas it was only 8.0% for the anti-Prdx1 antibody in 50 normal individual controls (11). In another study with HCC, only 2 of the 70 (2.9%) HCC sera showed a positive response to Prdx1, and 1 of 70 (1.4%) normal human sera was positive to Prdx1, as detected by ELISA with phage-expressed Prdx1 protein as the coating antigen (29). In the present study, we also tested the anti-Prdx1 antibody in HCC. The positive rate of anti-Prdx1 antibody was only 3.8% (3/78) and 2.4% (2/82) in normal human sera, respectively (data not shown). This preliminary data also suggest that the autoantibody against Prdx1 may be used as a potential biomarker in certain types of cancer but not for all types of cancer.

Although it is still unclear how autoantibodies are developed by the human immune system, many studies have demonstrated that autoantibody production is related to aberrant expression of autoantigens, such as the alteration of expression level and structural changes of cellular proteins (11,30). Autoantibodies can be detected in the sera of patients with autoimmune disease and in many tumors (31). In the present study, the titer of the autoantibody against Prdx1 in the sera from patients with ESCC was much higher than that in normal individuals. The positive rate of the autoantibody to Prdx1 was 13.0% in ESCC, and all the normal sera were negative when using the mean OD value plus 3 SD of the NHS group as a cutoff value. Taken together with the results from western blotting and IIF analysis, our data indicate that Prdx1 induces strong humoral autoimmune responses in some ESCC patients, suggesting that Prdx1 may be an ESCC-associated autoantigen, and the autoantibody against Prdx1 can be used as a potential serological biomarker in the immunodiagnosis of ESCC. The underlying mechanism of how this protein induces a humoral immune response in ESCC remains to be investigated.

## Figures and Tables

**Table I:** Frequency of an autoantibody to Prdx1 in human sera by ELISA.

Type of sera	No. tested	Autoantibody to PRDX1, n (%)
ESCC	68	9 (13.2)^a^
Normal	89	0 (0.0)

Cutoff value, means ± 3 SD of NHS;

a P<0.01 relative to NHS.

Prdx1, peroxiredoxin 1; ESCC, esophageal squamous cell carcinoma; NHS, normal human sera.

**Table II:** Expression profile of Prdx1 in ESCC, adjacent carcinoma and normal tissues including the score and intensity.

			Score and intensity						
			
				1			2		
					
Type of tissues	No. tested	Positive, n (%)	0	+	++	+++	+	++	+++
ESCC	16	16 (100)	0	2	2	1	4	3	4
Adjacent carcinoma	14	9 (64.3)	5	1	3	2	0	0	3
Normal	12	6 (50.0)	5	1	1	0	3	0	2

P<0.01, ESCC compared to normal tissue; P>0.05, adjacent carcinoma to normal tissue; P<0.05, ESCC to adjacent carcinoma. Scoring criteria: 0, 0% of the total cell population is positive; 1, 1–50% of the total cell population is positive; 2, 50–100% of the total cell population is positive. +, weak intensity; ++, mild intensity; +++, strong intensity. Prdx1, peroxiredoxin 1; ESCC, esophageal squamous cell carcinoma.

**Table III:** Expression profile of Prdx1 in paired ESCC, adjacent carcinoma and normal tissues.

	Tissues		
	
No.	ESCC	Adjacent	Normal
1	+	+	+
2	+	−	−
3	+	−	−
4	+	−	−
5	+	+	+
6	+	+	+
7	+	−	−

Cochran’s Q, 8.000, P<0.05. Prdx1, peroxiredoxin 1; ESCC, esophageal squamous cell carcinoma.

## References

[1] Lee SY, Jeoung D (2007). The reverse proteomics for identification of tumor antigens. J Microbiol Biotechnol.

[2] Fernández Madrid F, Tang N, Alansari H, Karvonen RL, Tomkiel JE (2005). Improved approach to identify cancer-associated autoantigens. Autoimmun Rev.

[3] Rhee SG (2006). Cell signaling. H_2_O_2_, a necessary evil for cell signaling. Science.

[4] Rhee SG, Chae HZ, Kim K (2005). Peroxiredoxins: a historical overview and speculative preview of novel mechanisms and emerging concepts in cell signaling. Free Radic Biol Med.

[5] Neumann CA, Cao J, Manevich Y (2009). Peroxiredoxin 1 and its role in cell signaling. Cell Cycle.

[6] Cao J, Schulte J, Knight A (2009). Prdx1 inhibits tumorigenesis via regulating PTEN/AKT activity. EMBO J.

[7] Hoshino I, Matsubara H, Akutsu Y (2007). Tumor suppressor Prdx1 is a prognostic factor in esophageal squamous cell carcinoma patients. Oncol Rep.

[8] Zhang J, Wang K, Zhang J, Liu SS, Dai L, Zhang JY (2011). Using proteomic approach to identify tumor-associated proteins as biomarkers in human esophageal squamous cell carcinoma. J Proteome Res.

[9] Kim YJ, Lee WS, Ip C, Chae HZ, Park EM, Park YM (2006). Prx1 suppresses radiation-induced c-Jun NH2-terminal kinase signaling in lung cancer cells through interaction with the glutathione S-transferase Pi/c-Jun NH2-terminal kinase complex. Cancer Res.

[10] Kim JH, Bogner PN, Baek SH (2008). Up-regulation of peroxiredoxin 1 in lung cancer and its implication as a prognostic and therapeutic target. Clin Cancer Res.

[11] Chang JW, Lee SH, Jeong JY (2005). Peroxiredoxin-I is an autoimmunogenic tumor antigen in non-small cell lung cancer. FEBS Lett.

[12] Yonglitthipagon P, Pairojkul C, Chamgramol Y (2012). Prognostic significance of peroxiredoxin 1 and ezrin-radixin-moesin-binding phosphoprotein 50 in cholangiocarcinoma. Hum Pathol.

[13] Li J, Yang ZL, Ren X (2013). ILK and PRDX1 are prognostic markers in squamous cell/adenosquamous carcinomas and adenocarcinoma of gallbladder. Tumour Biol.

[14] Quan C, Cha EJ, Lee HL, Han KH, Lee KM, Kim WJ (2006). Enhanced expression of peroxiredoxin I and VI correlates with development, recurrence and progression of human bladder cancer. J Urol.

[15] Riddell JR, Bshara W, Moser MT, Spernyak JA, Foster BA, Gollnick SO (2011). Peroxiredoxin 1 controls prostate cancer growth through Toll-like receptor 4-dependent regulation of tumor vasculature. Cancer Res.

[16] Chung KH, Lee DH, Kim Y (2010). Proteomic identification of overexpressed PRDX 1 and its clinical implications in ovarian carcinoma. J Proteome Res.

[17] Gordis L (1996). Assessing the validity and reliability of diagnostic and screening tests. Epidemiology.

[18] Das S, Otani H, Maulik N, Das DK (2006). Redox regulation of angiotensin II preconditioning of the myocardium requires MAP kinase signaling. J Mol Cell Cardiol.

[19] Kamata H, Honda S, Maeda S, Chang L, Hirata H, Karin M (2005). Reactive oxygen species promote TNFα-induced death and sustained JNK activation by inhibiting MAP kinase phosphatases. Cell.

[20] Wen ST, Van Etten RA (1997). The PAG gene product, a stress-induced protein with antioxidant properties, is an Abl SH3-binding protein and a physiological inhibitor of c-Abl tyrosine kinase activity. Genes Dev.

[21] Neumann CA, Krause DS, Carman CV (2003). Essential role for the peroxiredoxin Prdx1 in erythrocyte antioxidant defence and tumour suppression. Nature.

[22] Benhar M, Dalyot I, Engelberg D, Levitzki A (2001). Enhanced ROS production in oncogenically transformed cells potentiates c-Jun N-terminal kinase and p38 mitogen-activated protein kinase activation and sensitization to genotoxic stress. Mol Cell Biol.

[23] Barranco-Medina S, Lázaro JJ, Dietz KJ (2009). The oligomeric conformation of peroxiredoxins links redox state to function. FEBS Lett.

[24] Sirvent A, Benistant C, Roche S (2008). Cytoplasmic signalling by the c-Abl tyrosine kinase in normal and cancer cells. Biol Cell.

[25] Mu ZM, Yin XY, Prochownik EV (2002). Pag, a putative tumor suppressor, interacts with the Myc Box II domain of c-Myc and selectively alters its biological function and target gene expression. J Biol Chem.

[26] Hoskins ER, Hood BL, Sun M, Krivak TC, Edwards RP, Conrads TP (2011). Proteomic analysis of ovarian cancer proximal fluids: validation of elevated peroxiredoxin 1 in patient peripheral circulation. PLoS One.

[27] Hoshino I, Matsubara H, Hanari N (2005). Histone deacetylase inhibitor FK228 activates tumor suppressor Prdx1 with apoptosis induction in esophageal cancer cells. Clin Cancer Res.

[28] Chen MF, Keng PC, Shau H (2006). Inhibition of lung tumor growth and augmentation of radiosensitivity by decreasing peroxiredoxin I expression. Int J Radiat Oncol Biol Phys.

[29] Liu H, Zhang J, Wang S (2012). Screening of autoantibodies as potential biomarkers for hepatocellular carcinoma by using T7 phase display system. Cancer Epidemiol.

[30] Backes C, Ludwig N, Leidinger P (2011). Immunogenicity of autoantigens. BMC Genomics.

[31] Tan EM, Zhang J (2008). Autoantibodies to tumor-associated antigens: reporters from the immune system. Immunol Rev.

